# TARGETED SCREENING FOR LIVER FIBROSIS: IDENTIFYING HIGH-RISK ADULTS FOR TRANSIENT ELASTOGRAPHY EVALUATION

**DOI:** 10.1093/geroni/igae098.2513

**Published:** 2024-12-31

**Authors:** Amina Namrouti, Paulo Chaves

**Affiliations:** Florida International University College of Medicine, Miami, Florida, United States; Florida International University, Miami, Florida, United States

## Abstract

Non-alcoholic fatty liver disease (NAFLD) poses a significant clinical concern, impacting over 30% of the general population and leading to potentially reversible liver fibrosis, cirrhosis, and physical frailty. Transient elastography (TE) offers a non-invasive screening tool, measuring liver stiffness as an indicator of hepatic fibrosis, guiding subsequent biopsy decisions. However, TE’s availability and cost hinders its widespread use. Clinical practice relies on individual indicators like obesity for TE testing referrals. We aim to assess the accuracy of traditional indicators and explore whether combining readily available indicators in primary care settings could enhance screening accuracy. We conducted a cross-sectional analysis using National Health and Nutrition Examination Survey (NHANES) 2017-2020 data, involving adults aged ≥ 40 years without excessive alcohol consumption, cirrhosis, or hepatitis B/C. We evaluated liver stiffness measurements (LSM) >7pKa by TE as the outcome. Various predictors, including systemic diseases, examination findings, and laboratory tests, were assessed using stepwise logistic regression with sampling weights. Area under the receiver operating curve (AUC-ROC) was utilized to compare predictive accuracy across models. Results showed modest accuracy of individual indicators with AUC-ROC of 52%. The final model, incorporating diabetes, BMI>30 kg/m2, HR, A1c, FIB-4>3.25, APRI>0.5, and AST>40 IU/l, yielded an improved AUC-ROC of 74%. Combining primary care indicators enhanced identification of at-risk adults for abnormal LSM, compared to relying solely on individual indicators. Further research is necessary to determine the optimal combination of indicators for identifying individuals at risk of liver fibrosis who may benefit from TE screening.

